# Differential Impact of Malnutrition on Health Outcomes Among Indigenous and Non-Indigenous Adults Admitted to Hospital in Regional Australia—A Prospective Cohort Study

**DOI:** 10.3390/nu10050644

**Published:** 2018-05-19

**Authors:** Natasha Morris, Simon Stewart, Malcolm Riley, Graeme Maguire

**Affiliations:** 1 Monash University, Department of Epidemiology and Preventive Medicine; Baker Heart and Diabetes Institute; Melbourne 3004, Australia; 2 The University of Melbourne, Department of Nursing, Melbourne 3052, Australia; 3 The Queen Elizabeth Hospital, Cardiology Unit, Adelaide 5112, Australia; simon.stewart64@gmail.com; 4 CSIRO Health and Biosecurity, Adelaide 5000, Australia; malcolm.riley@csiro.au; 5 Western Health, General Internal Medicine, Melbourne 3001, Australia

**Keywords:** malnutrition, Indigenous Australians, subjective global assessment, Australia, survival

## Abstract

The burden of malnutrition in Indigenous people is a major health priority and this study’s aims are to understand health outcomes among Indigenous and non-Indigenous patients. This cohort study includes 608 medical inpatients in three regional hospitals. Participants were screened for malnutrition using the Subjective Global Assessment tool. Hospital length of stay, discharge destination, 30-day and six-month hospital readmission and survival were measured. Although no significant difference was observed between Indigenous participants who were malnourished or nourished (*p* = 0.120), malnourished Indigenous participants were more likely to be readmitted back into hospital within 30 days (Relative Risk (*RR*) 1.53, 95% CI 1.19–1.97, *p* = 0.002) and six months (*RR* 1.40, 95% Confidence Interval (CI) 1.05–1.88, *p* = 0.018), and less likely to be alive at six months (*RR* 1.63, 95% CI 1.20–2.21, *p* = 0.015) than non-Indigenous participants. Malnutrition was associated with higher mortality (Hazards Ratio (*HR*) 3.32, 95% CI 1.87–5.89, *p* < 0.001) for all participants, and independent predictors for six-month mortality included being malnourished (*HR* 2.10, 95% CI 1.16–3.79, *p* = 0.014), advanced age (*HR* 1.04, 95% CI 1.02–1.06, *p* = 0.001), increased acute disease severity (Acute Physiology and Chronic Health Evaluation score, *HR* 1.03, 95% CI 1.01–1.05, *p* = 0.002) and higher chronic disease index (Charlson Comorbidity Index, *HR* 1.36, 95% CI 1.16–3.79, *p* = 0.014). Malnutrition in regional Australia is associated with increased healthcare utilization and decreased survival. New approaches to malnutrition-risk screening, increased dietetic resourcing and nutrition programs to proactively identify and address malnutrition in this context are urgently required.

## 1. Introduction

Malnutrition is a highly prevalent problem in adult hospital patients in both low and high-income countries [1,2,3]. This is of clinical and public health significance given that recent cohort studies demonstrate a direct relationship between malnutrition and adverse health outcomes [2,4,5]. This includes increased healthcare utilization through longer lengths of hospital stay, frequent hospital admissions, and increased mortality when compared with nourished patients. In Australia, nearly a one-third of adult patients are reported to be malnourished [2]. However, given the diverse and dispersed Australian population and presence of high-risk/vulnerable communities beyond major population centers [6,7], we recently examined the pattern and prevalence of malnutrition among a representative cohort of 608 inpatients admitted to three hospitals located in regional Australia [8]. Overall, we found a higher than expected prevalence of malnutrition (41.7%), which was largely driven by a higher prevalence of malnutrition among Indigenous compared to non-Indigenous Australians [8]. This key differential was most evident amongst Indigenous Australians residing in Central Australia where 57 per cent of Indigenous versus 33% of non-Indigenous Australians were found to be malnourished [8]. Our initial findings of a higher prevalence of malnutrition among Indigenous people confirmed the potential role of malnutrition in contributing to persistent disparities in health outcomes; even among those who have accessed hospital care [8]. In a follow-up of our study cohort, we sought to determine the subsequent impact of malnourishment on short-to-medium health outcomes in the same cohort; hypothesizing that malnourishment would have a greater negative impact among Indigenous Australian people.

## 2. Materials and Methods

### 2.1. Study Design, Setting and Participants

**Figure 1:**
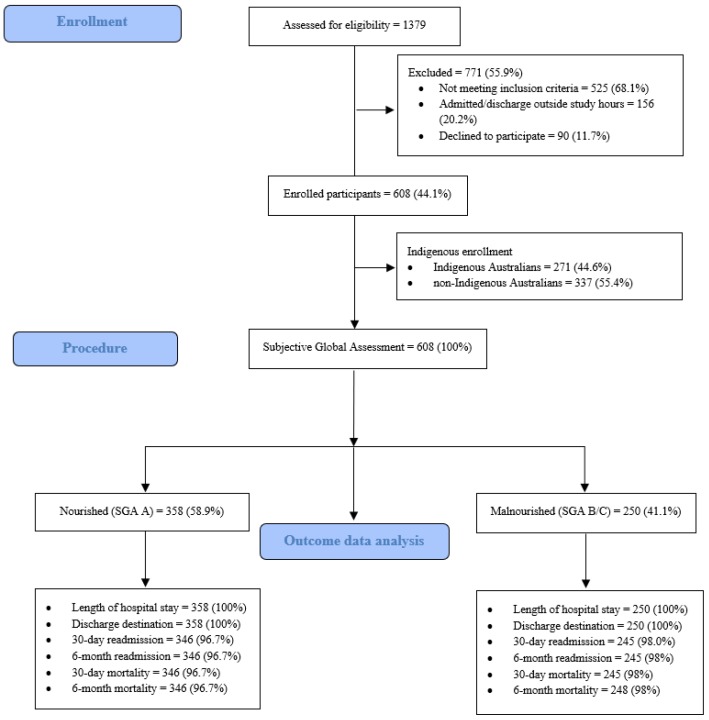
Study recruitment pathway and data available for final outcome analysis.

The study rationale and design and initial findings of the Indigenous Australian Malnutrition Project have been reported previously [8,9]. This prospective cohort study used convenience sampling and was conducted in two regional hospitals in the Northern Territory and one regional hospital in Far North Queensland. Figure 1 outlines the recruitment of 608 participants enrolled into this study between February 2015 and September 2015.

### 2.2. Nutrion Status and Disease Serverity Outcomes

As shown in Figure 1, all participants were assessed for malnutrition using the validated Subjective Global Assessment (SGA) tool within 72 h of admission by two study investigators (a Registered Nurse or an Accredited Dietitian). As per the SGA criteria, participants with an SGA of “A” were classified as “nourished” and participants with an SGA of B (mild-moderate malnutrition) or C (severe malnutrition) were classified as “malnourished” [10]. Acute and chronic disease severity (Acute Physiology and Chronic Health Evaluation (APACHE) III and Charlson Comorbidity Index (CCI)) were calculated in all study participants [11,12]. We used the APACHE III calculator in this study as the APACHE tools are the only prognostic calculator that assess acute physiologic distress including serum biochemistry and hematology [11]. To gain a better understanding of participants nutritional status, body mass index (BMI) was calculated in 579/608 (95.2%) of study participants.

### 2.3. Health Outcomes

Several short and medium-term health outcomes were examined according to participants nutritional status (nourished or malnourished) or their Indigenous status (Indigenous Australian or non-Indigenous Australian). Selected outcomes of interest as shown in Figure 1 included length of hospital index admission (date and time of admission to date and time of hospital separation); inpatient survival (alive or dead); hospital discharge destination (usual residential address); 30-day and six-month hospital readmission; and all-cause survival status from the point of time of hospital separation of the index admission. Length of hospital stay and discharge destination were calculated and determined in all study participants and data for 30-day and six-month hospital readmission were available as outlined in Figure 1.

Outcome data were collected and correlated with each hospitals’ medical record electronic databases by the study’s investigators during the February 2015 to September 2015 study period and cross-checked at the point of six months (March 2016) following the last participant enrolment in Far North Queensland (September 2015). Participant survival data were independently collected by hospitals’ medical record reporting officers and then confirmed by the first author (NM) using each Territory and State’s Births, Deaths and Marriages registrars [13,14].

### 2.4. Statistical Analysis

Data analysis was undertaken using Stata Release 15.1 (StataCorp, College Station, TX, USA). Descriptive data summarizing participants’ characteristics and health outcomes were summarized using standard univariate techniques and reported as frequencies and percentages with 95 per cent confidence intervals (95% CI), medians with first and third interquartile ranges (IQR) after assessing the format and distribution of data. Unadjusted comparisons between nutrition status and Indigenous status were undertaken using chi-square tests (*X*^2^) for categorical data, and Mann-Whitney *U* test for continuous non-parametric data. Further unadjusted comparisons measuring Indigenous versus non-Indigenous participants’ health outcomes and nutrition status were undertaken by using Wilcoxon rank-sum test. A *p* value < 0.05 was taken to indicate statistical significant and all tests were two-sided.

Survival analyses for mortality are presented as Kaplan–Meir curves and analyzed using the log-rank test. Multivariable linear, logistic and Cox proportional hazard modelling were utilized using a back stepwise approach to identify independent factors associated with health outcome measures (length of hospital index admission, six-month hospital readmission, and six-month mortality) that were identified as significantly different in the univariate analyses. These factors included age (years); nutrition status (nourished or malnourished); APACHE III and CCI scores. All factors were included in the first model using bivariate analysis with a *p* value < 0.1. Factors with a *p* value ≥ 0.05 were progressively removed from the models starting with variables that contributed least to predictive modelling. Final models were limited to predictive factors with significant coefficients (*p* < 0.05).

### 2.5. Ethics Approval

Approval for this study was granted by Monash University (CF14/3350 2014001787); Central Australia (HREC-14-256); Menzies School of Health Research (HREC 2014-2282); and Far North Queensland (HREC/141QCH/86-927) Human Research Ethics Committees.

## 3. Results

### 3.1. Participant Characteristics

The characteristics of study participants according to their nutrition status are summarized in Table 1. The overall prevalence of malnutrition in this study cohort was (41.1%, 95% CI 37.2–45.1). Indigenous participants were more likely to be malnourished than non-Indigenous participants (Odds Ratio (*OR*) 1.45, 95% CI 1.03–2.04, *p* = 0.024) and malnourished participants were significantly older than nourished patients by 4.2 years. Malnourished participants had a significantly lower median BMI than nourished patients (21.6 kg/m^2^ versus 28.8 kg/m^2^, respectively), and when compared to nourished participants, malnourished participants had a higher median APACHE III score (34 versus 27, respectively) and higher mean CCI score (2.7 versus 2.0, respectively) than nourished participants. No significant different differences were observed between participants gender and nutrition status.

### 3.2. Health Outcomes for Nourished and Malnoursihed Participants

Health outcomes according to each participant’s nutritional status at baseline are summarized in Table 2. Although there was no difference was observed in respect to discharge destination at the time of hospital separation, length of hospital stay among malnourished participants was nearly two days longer than nourished participants. Likewise, although no significant difference was observed in the total number of hospital readmissions at six months, malnourished participants were more likely to be readmitted into hospital at 30-day (*RR* 1.51, 95% CI 1.25–1.82) and at six months (*RR* 1.48, 95% CI 1.21–1.81). While in-hospital mortality did not vary by nutritional status, mortality at 30 days (*RR* 1.68, 95% CI 1.24–2.27) and at six months (*RR* 1.78, 95% CI 1.45–2.19) were significantly higher in malnourished than nourished participants.

### 3.3. Health Outcomes Among Indigenous and Non-Indigenous Australian Participants

As summarized in Table 3, no significant differences were observed between Indigenous and non-Indigenous participants and their length of hospital index stay, participants discharge destination, 30-day hospital readmissions and six-month survival. Overall however, Indigenous Australians were more likely to be readmitted to hospital within six months (*RR* 1.39, 95% CI 1.15–1.68) and have a higher total number of hospital readmissions at six months and in contrast, non-Indigenous participants were less likely to survive at 30 days (*RR* 1.52, 95% CI 1.18–1.94). 

A summarized in Table 4, nutritional status appeared to modulate some of these key differentials in health outcomes. For example, Indigenous Australians who were malnourished were more likely to be readmitted back into hospital within 30-day (*RR* 1.53, 95% CI 1.19–1.97) and six months (*RR* 1.40, 95% CI 1.05–1.88) and were less likely to be alive at six months than nourished Indigenous participants (*RR* 1.63, 95% CI 1.20–2.21). Likewise, non-Indigenous Australians who were malnourished were also likely to be readmitted back into hospital at 30 days (*RR* 1.46, 95% CI 1.10–1.95) and six months (*RR* 1.53, 95% CI 1.15–2.05) and were less likely to survive at six months (*RR* 2.07, 95% CI 1.56–2.75). In addition, non-Indigenous participants who were malnourished were also likely to have longer length of hospital index stay, more frequent six-month hospital readmissions and less likely to survive at 30 days than non-Indigenous participants who were nourished (*RR* 2.13, 95% CI 1.53–3.00).

### 3.4. Independment Factors Associated with Healthcare Ultisation and Decreased Survival

Factors independently associated with index length of hospital admission, six-month subsequent hospital readmission and six-month mortality are presented in Table 5. We tested the hypothesis that malnutrition would be an independent predictor of increased risk of all cause readmission and decreased survival in Indigenous and non-Indigenous participants at six-month follow-up. We found that being malnourished conveys the same negative impact for six-month hospital readmission for both Indigenous and non-Indigenous participants, but for Indigenous Australian participants, being malnourished was an independent predictor for mortality at six months. Similarly, for both Indigenous and non-Indigenous participants, increased acute disease severity score (APACHE III) was an independent predictor for increased length of hospital stay; and an increased acute disease severity score (APACHE III) and chronic comorbidity score (CCI) were both predictors for six-month hospital readmission and mortality at six months.

**Figure 2:**
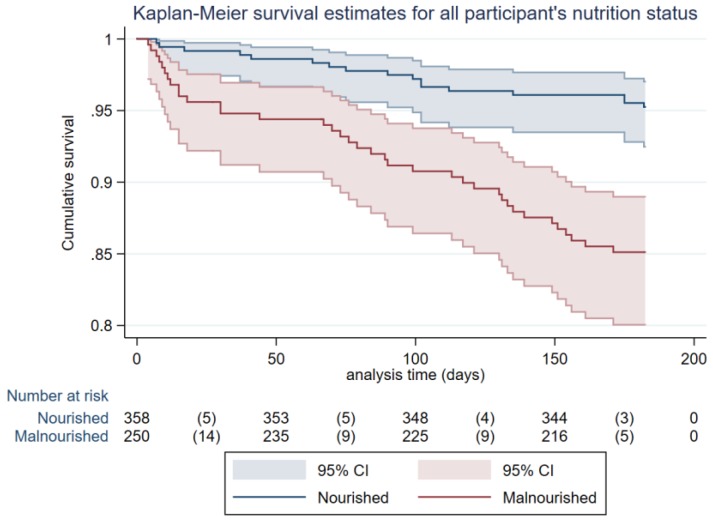
Unadjusted survival for Indigenous and non-Indigenous participants to six months stratified by nutritional status (nourished or malnourished), (Log rank, *p* < 0.001; *HR* 3.32, 95% CI 1.87–5.89).

**Figure 3:**
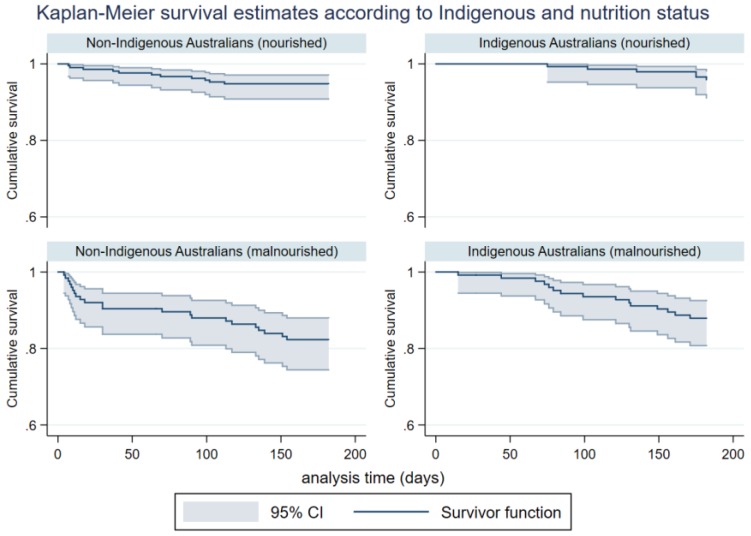
Unadjusted survival for Indigenous participants to six months stratified by nutritional status (Log rank, *p* = 0.013, *HR* 3.12, 95% CI 1.21–8.04), and unadjusted survival for non-Indigenous participants to six months stratified by nutritional status (Log rank, *p* = 0.001, *HR* 3.64, 95% CI 1.76–7.50).

Unadjusted mortality to six months illustrate higher mortality in malnourished patients when stratified by nutrition status (see Figure 2). Likewise, unadjusted mortality to six months was significantly higher in both Indigenous and non-Indigenous Australians who were malnourished (see Figure 3).

## 4. Discussion

This cohort study is the first to examine the subsequent impact of malnutrition and measure health outcome differences between hospitalized Indigenous and non-Indigenous people. Overall, we found that malnutrition was a key driver of increased health care utilization and increased risk of mortality for both Indigenous and non-Indigenous participants, highlighting a need for increased dietitian resourcing and urgent community and hospital-based nutrition programs. For example, for every 100 patients who are malnourished, 64.5% will be readmitted back into hospital within six months compared to 47.7% of nourished patients and 14.3% of malnourished patients will die at six months compared to 4.0% of patients who are nourished. Likewise, for every 100 Indigenous patients who are malnourished 70.2% will be readmitted back into hospital within six months compared to 58.7% malnourished non-Indigenous patients. However, 16.5% of non-Indigenous malnourished patients will die within six months compared to 12.1% of malnourished Indigenous patients. The higher attributable risk for six-month mortality in malnourished non-Indigenous patients when compared to Indigenous patients is likely due to our early study finding that non-Indigenous patients were significantly older than non-Indigenous patients by nearly 12 years (*p* < 0.001) [7], highlighting the need for early nutrition assessment in all patients with advanced age. However, we did find that malnutrition was also an independent predictor for six-month mortality for Indigenous Australians confirming that malnutrition is a significant problem for Indigenous Australian patients.

Furthermore, we found that readmission rates for Indigenous Australian participants were significantly higher than non-Indigenous participants at 30 days (27.0% versus 22.1%, respectively) and at six months (62.6% versus 48.0%, respectively). This finding is comparable to other studies differentiating health care utilization in Indigenous and non-Indigenous Australians. Whyatt et al. conducted a longitudinal cohort study from 2002 to 2014 comparing rates of hospital utilization in Indigenous and non-Indigenous Australians with chronic diseases [15]. Whyatt et al. found that while inpatient length of stay in Indigenous Australian patients were similar to non-Indigenous patients (as we found in our study), Indigenous Australians had much higher rates of Emergency Department presentations, were more likely to be admitted as an inpatient, and were also younger than non-Indigenous Australians [15]. Common factors found in our study and other studies predicting increased healthcare utilization for Indigenous and non-Indigenous Australian patients include the presence of chronic disease and more telling, severity of disease. Indigenous Australian patients residing in remote regions like Central Australia or people residing in very remote regions up to 1000 km from the nearest hospital, are more likely to be admitted to hospital than non-Indigenous Australians who are more likely to live in inner or outer regional areas with closer access to community and hospital health services [16].

Given the high proportion of malnutrition we found particularly amongst Indigenous participants, and the subsequent impact of malnutrition for both Indigenous and non-Indigenous participants, these data identify malnutrition as a high priority target for addressing these adverse health outcomes. For example, the national average cost of an overnight stay in an Australian acute care hospital bed is $2074 [17], and in our study, this represented just over $3700 for every patient who was malnourished. We found that rates of hospital readmission were much higher when compared to other longitudinal studies in Australian hospitals. For example, in a large retrospective study measuring hospital readmission rates over a 5-year period in a large general medicine tertiary hospital in South Australia, the 28-day readmission rate was 10.8 per cent [18]. In our study, the total readmission rate at 30 days for all study participants was higher at 24.3% and as high as 32.6% for malnourished participants demonstrating increased healthcare utilization in regional settings and malnutrition being a significant contributing factor.

Although there are several important community-based nutrition intervention programs [19], there is an urgent need to explore strategies for nutrition management for people with chronic disease. This requires a more considered and strategic approach to detecting malnutrition in patients who are admitted to hospital. While screening patients for malnutrition within 24 hours is recommended practice [20], the reliability of malnutrition screening varies greatly depending on the tool being used and how screening is being conducted [21]. Detecting malnutrition early in a patient’s hospital admission is imperative to avoid adverse health outcomes. Further studies are required to consider using acute disease severity scores such as an adapted APACHE scoring system for medical inpatients as our study found that increased healthcare utilization for patients who were malnourished as well as Indigenous patients is attributable to increased acute and chronic disease physiologic scores. Likewise, due to the chronic nature of malnutrition in adults, community-based screening in community health centers should be implemented as part of routine health checks in patients with a chronic disease. Subsequently, there is an urgent need for increased dietetic resources both in the community and in hospitals.

One of the major limitations of our study includes the relatively short follow-up time at six months from patients’ index hospital admission. Lim et al. measured mortality in malnourished and nourished patients at three years from their initial index admission found that nearly half (48.5%) of the patients with malnutrition had died compared to just under 10 per cent of nourished patients (RR 4.8, 95% CI 3.7–6.5, <0.001) [4]. This finding highlights the necessity for longer-term follow-up studies to measure the true long-term impact of malnutrition especially for Indigenous Australian patients where the rate of malnutrition is higher and disease severity higher. This study is generalizable to other high-income countries where health disparities between Indigenous and non-Indigenous populations exist but when comparing our study’s findings with other malnutrition-related health outcome studies, caution should be exercised due to the unique geographical location and health services where our study was conducted.

## 5. Conclusions

This study has confirmed that malnutrition for both Indigenous and non-Indigenous patients admitted into regional hospital settings results in greater healthcare utilization and increased risk of mortality than patients who are nourished. In our study, malnutrition is not only associated with increased hospital readmission and decreased survival, but acute and chronic disease severity in our study’s participants were predicators for increased length of hospital stay and/or hospital readmission and mortality. As an adjunct to malnutrition screening, further research is required to explore the use of acute physiologic scores (i.e., a modified APACHE scoring system) for medical inpatients to aide in detecting malnutrition in patients with an acute illness or with severe chronic disease. Further research is also required to understand the financial burden of malnutrition on health care services through increased length of hospital stays and hospital readmissions. It is also important that future research focuses on chronic-disease related malnutrition in adults residing in the community and the evaluation of nutritional intervention strategies as part of secondary and tertiary prevention management. 

## Figures and Tables

**Table 1:** Characteristics of study participants according to their nutrition status (nourished or malnourished).

	All *n =* 608		Nourished (SGA A) *n* (%) = 358 (58.9)		Malnourished (SGA B or C) *n* (%) = 250 (41.1)		*p* Value
Age, median (IQR)	61.6 (49.1–72.2)		59.2 (48.0–70.5)		63.4 (50.9–73.3)		0.009
Gender (female), *n* (%), 95% CI	294 (48.4)	44.3–52.4	179 (50.0)	44.7–55.3	115 (46.0)	39.7–52.4	0.331
Indigenous status (Indigenous Australian) *n* (%) 95% CI	271 (44.6)	40.6–48.6	146 (53.9)	47.7–59.9	125 (46.1)	40.1–52.3	0.024
BMI (kg/m^2^), median (IQR)	26.3 (21.6–31.2)		28.8 (25.4–34.2)		21.6 (18.8–25.4)		<0.001
APACHE III, median (IQR)	30 (20–30)		27 (19–36)		34 (24–44)		<0.001
CCI, median (IQR)	2 (1–3)		2 (1–3)		2 (1–4)		<0.001

**Table 2:** Health outcomes according to participants nutrition status (nourished or malnourished).

	All *n =* 608		Nourished (SGA A) *n* (%) = 358 (58.9)		Malnourished (SGA B or C) *n* (%) = 250 (41.1)		*p* Value
Length of stay median (IQR)	4.7 (2.6–9.1)		4.1 (2.2–7.7)		5.9 (2.9–11.1)		<0.001
Discharge destination *n* (%), 95% CI							
Usual residential address	503 (82.7)	79.5–85.7	291 (81.3)	76.9-85.2	212 (84.8)	79.7–89.0	0.259
Hospital readmissions *n* (%), 95% CI							
30 days	145/591 (24.3)	20.9–8.0	64–346 (18.4)	14.5–22.9	81/245 (32.6)	26.9–38.8	<0.001
6 months	323/591 (54.2)	50.1–58.2	165/346 (47.4)	42.1–52.8	158/245 (63.7)	57.4–69.7	<0.001
Mortality *n* (%), 95% CI							
30 days	22/591 (3.7)	2.3–5.5	4/346 (1.2)	0.3–2.9	12/245 (4.9)	2.6–8.4	0.006
6 months	55/591 (9.2)	7.0–11.8	14/346 (4.0)	2.2–6.7	35/245 (14.3)	10.2–19.3	<0.001

**Table 3:** Health outcomes according to participants Indigenous status.

	Indigenous Australian *n* (%) = 271 (44.6)		Non-Indigenous Australian *n* (%) = 337 (55.4)		*p* Value
Length of stay, median (IQR)	5.0 (2.3–9.0)		4.6 (2.5–9.1)		0.186
Discharge destination *n* (%), 95% CI					
usual residential address	217 (80.1)	74.8–84.7	286 (84.9)	80.6–88.5	0.120
Hospital readmissions *n* (%), 95% CI					
30 days	73 (27.0)	21.8–32.8	72 (22.1)	17.7–27.1	0.195
6 months	169 (62.6)	56.5–68.4	154 (48.0)	42.4–53.6	<0.001
Mortality *n* (%), 95% CI					
30 days	3 (1.1)	0.2–3.2	13 (4.0)	2.2–6.8	0.028
6 months	21 (7.8)	4.9–11.6	28 (8.7)	5.9–12.4	0.678

**Table 4:** Health outcomes according to participants Indigenous and nutrition status.

	Indigenous Australians *n* (%) = 271 (44.6)		*p* Value	Non-Indigenous Australians *n* (%) = 337 (55.4)		*p* Value
	Nourished *n* (%) = 146 (53.9)	Malnourished *n* (%) = 125 (46.1)		Nourished *n* (%) = 212 (62.9)	Malnourished *n* (%) = 125 (37.1)	
Length of stay median (IQR)	4.8 (2.3–8.1)	5.8 (2.8–10.7)	0.120	3.8 (2.0–6.9)	6.2 (2.9–12.1)	0.001
Discharge destination (usual address) *n* (%), 95% CI	109 (74.7) 66.8–81.5	108 (86.4) 79.1–91.9	0.016	182 (85.8) 80.4–90.2	104 (83.2) 75.5–89.3	0.512
Hospital readmissions *n* (%), 95% CI						
30 days	28 (19.2) 13.1–26.5	45 (36.3) 27.8–45.4	0.002	36 (18.0) 12.9–24.0	36 (29.8) 21.2–38.7	0.014
6 months	82 (56.2) 47.7–64.4	87 (70.2) 61.3–78.0	0.018	83 (41.5) 34.6–48.7	71 (58.7) 49.4–67.5	0.003
Mortality *n* (%), 95% CI						
30 days	1 (0.7) 0.02–3.8	2 (1.6) 0.2–5.7	0.469	3 (1.5) 0.3–4.3	10 (8.3) 4.0–14.7	0.003
6 months	6 (4.1) 1.5–8.7	15 (12.1) 6.9–19.2	0.015	8 (4.0) 1.7–7.7	20 (16.5) 10.4–24.4	<0.001

**Table 5:** Independent predictors of increased length of stay, and 6-month hospital readmission & mortality.

	All *n* = 608			Indigenous Australian *n* = 271 (44.6%)	Non-Indigenous Australian *n* = 337 (55.4%)				
Length of hospital stay	Coef	95% CI	*p* value	Coef	95% CI	*p* value	Coef	95% CI	*p* value
APACHE III	15.0	7.8–22.3	<0.001	12.1	2.2–22.0	0.017	17.1	6.6–27.5	0.001
Psuedo *R*^2^ %	2.7			2.1	3.0				
Six-month readmission	OR	95% CI	*p* value	OR	95% CI	*p* value	OR	95% CI	*p* value
Malnourished (SGA B/C)	1.62	1.13–2.31	0.008	1.75	1.05–2.94	0.033	1.71	1.07–2.74	0.026
Indigenous Australian (yes)	1.60	1.12–2.27	0.009	-	-	-	-	-	-
APACHE III	1.01	1.00–1.03	0.045	-	-	-	-	-	-
CCI	1.23	1.10–1.37	<0.001	1.23	1.11–1.49	0.001	1.23	1.10–1.47	0.001
Psuedo *R*^2^ (%)	6.8			5.1	4.5				
Six-month mortality	HR	95% CI	*p* value	HR	95% CI	*p* value	HR	95% CI	*p* value
Malnourished (SGA B/C)	2.10	1.16–3.79	0.014	2.72	1.04–7.10	0.041	-	-	-
Age (years)	1.04	1.02–1.06	0.001	-	-	-	-	-	-
APACHE III	1.03	1.01–1.05	0.002	-	-	-	1.06	1.03–1.08	<0.001
CCI	1.36	1.16–3.79	0.014	1.48	1.27–1.74	<0.001	1.48	1.28–1.71	<0.001
Psuedo *R*^2^ (%)	24.1			16.6	33.2				
